# The Evolution of Match Running Performance in the Top Two Spanish Soccer Leagues: A Comparative Four-Season Study

**DOI:** 10.3390/jfmk10010027

**Published:** 2025-01-10

**Authors:** Tomás García-Calvo, David Lobo-Triviño, Javier Raya-González, Roberto López del Campo, Ricardo Resta, Eduard Pons, José Carlos Ponce-Bordón

**Affiliations:** 1 Faculty of Sport Sciences, University of Extremadura, 10003 Caceres, Spain; tgarciac@unex.es (T.G.-C.); joponceb@unex.es (J.C.P.-B.); 2 Research Group on Sport and Physical Education for Personal and Social Development, Faculty of Education Sciences and Psychology, University of Cordoba, 14071 Cordoba, Spain; rayagonzalezjavier@gmail.com; 3 Department of Competitions and Mediacoach, LaLiga, 28043 Madrid, Spain; rlopez@laliga.es (R.L.d.C.); rresta@laliga.es (R.R.); 4 Sport Performance Area, FC Barcelona, 08028 Barcelona, Spain; edu.pons.a@gmail.com

**Keywords:** football, physical demands, match analysis, longitudinal, sprinting

## Abstract

**Objectives**: This study uniquely examines the evolution of match running performance in official matches over four consecutive seasons (2019/2020–2022/23) within Spain’s top two professional soccer leagues (LaLiga). By analyzing differences between competitive league standards (First Division vs. Second Division), this research provides critical insights into how competition levels influence physical performance trends over time. **Methods**: A total of 6784 match observations were recorded from 95 teams competing in the First and Second Divisions (LaLiga). Performance metrics included total distance (TD), high-speed running (HSR, >21 km·h^−1^), very high-speed running (VHSR, 21.1–24 km·h^−1^), sprint distance (Sprint, >24 km·h^−1^), the number of HSR efforts (Nº. Sprints > 21 km·h^−1^), and sprint efforts (Nº. Sprints > 24 km·h^−1^), all analyzed using a computerized tracking system (TRACAB, ChyronHego, New York, NY, USA). **Results**: The primary findings indicated significantly higher match running performance in First Division matches compared to Second Division matches across all variables (*p* < 0.001). Furthermore, match running performance significantly increased over the four seasons in both leagues, with notably higher values during the 2021/22 and 2022/23 seasons for all physical performance metrics (*p* < 0.001). **Conclusions**: This study contributes to understanding the temporal evolution of soccer match performance across competition levels. The findings emphasize the importance of periodic performance monitoring and adapting training loads increasing high-intensity distances to align with escalating physical demands in modern soccer, offering valuable guidance for coaches and performance analysts.

## 1. Introduction

In modern soccer, tracking technologies such as GPS devices and video tracking systems allow for the comprehensive collection of match running performance data—the physical output of players during matches, measured through various metrics [1,2]. This technological advancement has significantly increased research interest in match running performance and its evolution over time, highlighting a notable rise in match intensity as players cover greater distances at higher speeds [3]. Additionally, studies suggest that soccer players’ physical profiles have evolved in recent years [3,4], emphasizing the need to adapt training periodization to meet modern and future match demands [5]. The growing number of matches and match density further underscore this evolution [6,7,8]. Given these changes, including increased high-speed running (HSR) and higher match intensity, an updated analysis of match running performance is essential.

Differences in match running performance across competitive leagues have been widely studied, but results remain inconsistent [9]. A key debate is whether higher-standard leagues require players to perform more HSR compared to lower-standard leagues. For example, some studies found that players in the lower tiers of English leagues covered greater total distance (TD) and HSR than those in the top tier [9,10]. In contrast, Mohr et al. [11] reported that elite Italian players covered 28% more HSR and 58% more sprinting distance than sub-elite Danish players. Similarly, Norway’s top league players recorded greater sprint distances than those in lower divisions [12]. Recent findings from the Spanish First Division also revealed higher high-intensity distances compared to the Second Division [13]. These contradictions may arise from differences in player characteristics and playing styles across countries [14,15]. Therefore, it is crucial to analyze match demands within individual leagues over time to understand both league-level differences and performance trends [3].

The evolution of match running performance has also been extensively investigated [16]. Research indicates a decline in TD covered alongside an increase in HSR and sprint distances over the years [4,13,17]. A systematic review of European leagues over the past two decades supports this trend [3]. However, recent studies of the English Premier League and Russian Premier League reported an overall increase in TD [18,19], suggesting that match demands might be shifting again. Another important consideration is the continuous increase in the high-intensity distance covered by soccer players [20,21,22]. Recent studies have shown that players cover greater distances at high intensities and in HSR metrics during matches [23]. Consequently, studies reporting distances covered at 24–28 km·h^−1^ or maximum distances (i.e., >28 km·h^−1^) are becoming more common [24]. For instance, HSR and sprint running distances have ranged from 911 to 1063 m and 223 to 307 m, reflecting increases of approximately 29% and 50%, respectively [23]. Therefore, the increase in maximum distances should be carefully considered in training load monitoring [25], in injury prevention [26], and in identifying elite players in professional soccer [22].

The integration of advanced tracking technologies has improved the understanding of match running performance and contributed to enhancing players’ physical capacities over the last decade. However, this evolution has also raised the physical demands of the game. Importantly, the most recent season analyzed in the Spanish professional soccer leagues was 2019/20 [13]. Therefore, this study provides an updated analysis of match running performance in the Spanish First and Second Divisions, offering valuable insights for coaches, practitioners, and sport scientists. Therefore, this study aimed to (i) compare match running performance between the top two competitive levels of Spanish professional soccer and (ii) analyze and compare the evolution of match running performance between the First and Second Divisions over four consecutive seasons (2019/20–2022/23). We hypothesized that TD, high-intensity distances, and the number of HSR efforts would be higher in the First Division compared to the Second Division. Additionally, based on recent trends, we hypothesized that both TD and high-intensity distances would increase in both leagues over the four seasons analyzed.

## 2. Materials and Methods

### 2.1. Sample

The sample comprised 6784 match observations from 95 teams competing in the First (*n* = 3040 records) and Second (*n* = 3744 records) Spanish soccer leagues over four consecutive seasons (from 2019/20 to 2022/23). Each season included 760 team match observations from the First Division and 936 team match observations from the Second Division. The data represent total team values (i.e., all players who participated in the matches regardless of the minutes played, including starters, non-starters, and substitutes). The Spanish Professional Football League (LaLiga) provided the data, allowing access to the variables included in this investigation. In accordance with LaLiga’s ethical guidelines, this investigation does not include any information that could identify individual players (General Assembly of LaLiga, 2019). This study received approval from the Bioethics Committee of the first author’s university (application number 239/2019).

### 2.2. Procedure

Match running performance data were recorded using a multicamera computerized optical tracking system called TRACAB (ChryronHego VID, New York, NY, USA) and managed through the Mediacoach application (LaLiga, Madrid, Spain). This system, consisting of a series of super 4K High Dynamic Range cameras, records each player from multiple angles, providing real-time three-dimensional tracking (tracking data are recorded at 25 Hz). The validity and reliability of this system for the selected variables were previously investigated [27,28], reporting variations between devices close to zero (<5%) for TD, distance per minute, average speed, maximum speed, and walking and jogging and between 9% and 15% for running, intense running, and sprinting at low and high intensities. Also, very large correlations (r > 0.75) and high intraclass correlation coefficients (ICCs > 0.75) between the Mediacoach multicamera tracking system and the Global Positioning System were shown. Correlation results were evaluated as trivial (<0.10), small (0.10–0.30), moderate (0.30–0.50), large (0.50–0.70), very large (0.70–0.90), and nearly perfect (0.90–1.00) [29]. Additionally, ICCs results were evaluated as very low (<0.20), low (0.20–0.50), moderate (0.50–0.75), high (0.75–0.90), very high (0.90–0.99), and extremely high (>0.99) scores [30,31].

### 2.3. Study Variables

#### 2.3.1. Dependent Variables

Match running performance was divided into the following categories: total distance covered by teams in meters (TD), high-speed running distance (HSR, >21 km·h^−1^), very high-speed running distance (VHSR, 21.1–24 km·h^−1^), and sprinting speed running distance (sprint, >24 km·h^−1^). In addition, the number of HSR efforts (i.e., Nº. Sprints > 21 km·h^−1^) and sprint efforts (i.e., Nº. Sprints > 24 km·h^−1^) were registered. All efforts involving a minimum movement of one meter, sustained for at least one second, were included. Any recording at a speed of over 80% of the value of that category (i.e., >24 km·h^−1^) was considered as a single register.

#### 2.3.2. Independent Variables

Different contextual variables were included in this study. Concerning the standard leagues, the top two Spanish professional soccer leagues (i.e., First and Second Divisions) were included similarly to previous studies [13]. In addition, four consecutive seasons (i.e., from 2019/20 to 2022/23) were also analyzed. Finally, regarding the match halves, the dependent variables were also shown and analyzed by matches and separated by halves (first half and second half).

### 2.4. Statistical Analysis

All statistical analyses were performed using RStudio (version 2024.12.0+467) [32] with the lme4 package to calculate Linear Mixed Models (LMMs) [33]. The additional packages used included emmeans for estimated marginal means [34]. Given the hierarchical structure of the sample, with data organized longitudinally and nested within groups, LMMs were chosen as the most appropriate method for analyzing the data, as they have demonstrated robustness in handling unbalanced and repeated-measures data [35,36]. For example, match running performance variables in matches are nested into teams (i.e., each team has a record for every match they have participated in, and each match has two observations of each team). Thus, cross-classified multilevel models are suitable for data structures that are not purely hierarchical. Consequently, a general multilevel modeling strategy was applied, including fixed and random effects in different steps, following the approach by Heck and Thomas [35].

LMMs were used to analyze the effects of competitive standard leagues on player activity across seasons. A two-level hierarchy was modeled for the analysis. Match running performance variables (i.e., distances covered at different speed thresholds and HSR and sprint efforts) were included as dependent variables in the models; leagues (First and Second Divisions) and seasons (2019/20, 2020/21, 2021/22, and 2022/23) were the independent variables included as fixed effects. The team variable was included as a random effect in the analysis. The results were represented as coefficient and standard error (Coeff ± SE). Statistical significance was set at *p* < 0.05. Finally, eta squared (η^2^) was included to calculate the effect size (ES) of the model with the following ranges: 0.01: low; 0.06: middle; and 0.14: elevated. To quantify the magnitude of difference for all pairwise comparisons between both leagues, Cohen’s D was also conducted using the following thresholds for interpretation: 0.00–0.19: trivial; 0.20–0.59: small; 0.60–1.19: moderate; 1.20–1.99: large; and ≥2.00: very large [37] (see Table S1).

## 3. Results

### 3.1. Match Running Performance Comparison Between Standard Leagues

**Figure 1:**
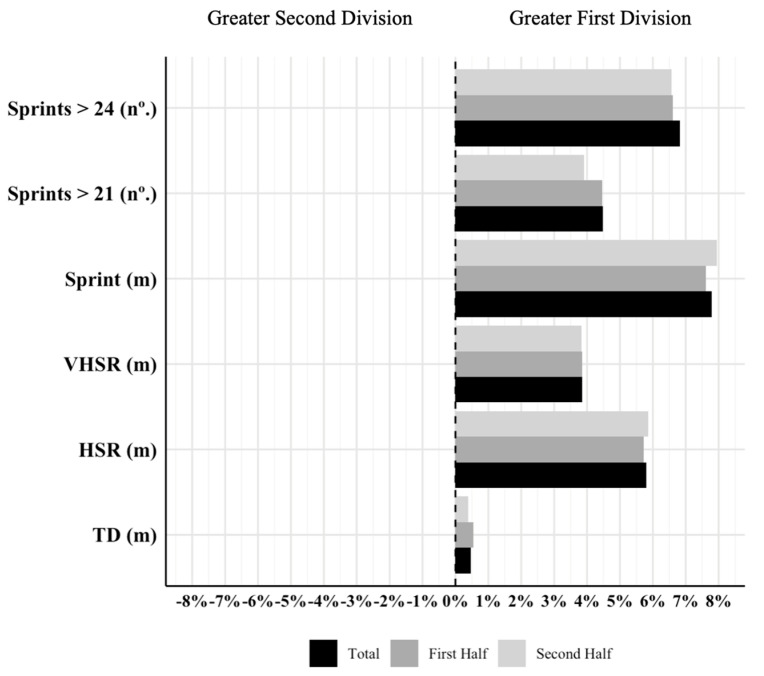
Percentage of change in match running performance between the First and Second Divisions. Note. m = meters; TD = total distance; HSR = high-speed running; VHSR = very high-speed running; Sprint = sprinting speed running distance.

Descriptive statistics for both competitive standard leagues over the four seasons are summarized in Table 1, while Figure 1 illustrates the differences in percentage from the First Division to the Second Division. First Division teams covered significantly greater TD, HSR, VHSR, sprint distance, Nº. Sprints > 21 km·h^−1^, and Nº. Sprints > 24 km·h^−1^ over the course of the match (*p* < 0.001) compared to teams in the Second Division. In the half-by-half analysis, First Division teams also covered significantly greater TD, HSR, VHSR, sprint distance, Nº. Sprints > 21 km·h^−1^, and Nº. Sprints > 24 km·h^−1^ during both the first (*p* < 0.001) and second halves (*p* < 0.001) compared to Second Division teams. Concerning the percentage of change, the differences in all variables analyzed were greater from the First Division to the Second Division.

### 3.2. Match Running Performance Comparison Between Seasons

Table 2 shows the evolution of TD and HSR in the First and Second Divisions over the four seasons. In the First Division, in the 2021/22 and 2022/23 seasons, teams covered significantly more TD compared to the 2019/20 (*p* < 0.001; ES: 0.47) and 2020/21 seasons (*p* < 0.001; ES: 0.38) and more HSR than the 2019/20 (*p* < 0.001; ES: 0.61) and 2020/21 seasons (*p* < 0.01; ES: 0.20). In the Second Division, in the 2021/22 season, teams covered significantly more TD compared to the 2019/20 (*p* < 0.001; ES: 0.79), 2020/21 (*p* < 0.001; ES: 0.68), and 2022/23 seasons (*p* < 0.01; ES: 0.18) and more HSR than the 2019/20 (*p* < 0.001; ES: 0.85), 2020/21 (*p* < 0.001; ES: 0.30), and 2022/23 seasons (*p* < 0.001; ES: 0.35). In general, small to moderate increases were seen in both soccer leagues for the TD and HSR variables between the 2019/20 and 2022/23 seasons (see Table S1).

Table 3 shows the evolution of VHSR and sprint distance in the First and Second Divisions over the four seasons. Considering the First Division, in the 2021/22 season, teams covered significantly more VHSR compared to the 2019/20 (*p* < 0.001; ES: 0.58) and 2020/21 seasons (*p* < 0.05; ES: 0.15) and more sprint distance than the 2019/20 (*p* < 0.001; ES: 0.53) and 2020/21 seasons (*p* < 0.01; ES: 0.21). When the Second Division was considered, in the 2021/22 season, teams also covered significantly more VHSR compared to the 2019/20 (*p* < 0.001; ES: 0.80), 2020/21 (*p* < 0.001; ES: 0.31), and 2022/23 seasons (*p* < 0.01 ES: 0.40) and more sprint distance than the 2019/20 (*p* < 0.001; ES: 0.76), 2020/21 (*p* < 0.001; ES: 0.25), and 2022/23 seasons (*p* < 0.001; ES: 0.25). Similarly, small to moderate increases were seen in both soccer leagues for VHSR and sprint variables between the 2019/20 and 2022/23 seasons (see Table S1).

Table 4 shows the evolution of high-speed running and sprinting speed running efforts in the First and Second Divisions over the four seasons. Considering the First Division, in the 2021/22 season, teams performed significantly more Nº. Sprints > 21 compared to the 2019/20 (*p* < 0.001; ES: 0.57) and 2020/21 seasons (*p* < 0.05; ES: 0.13) and more Nº. Sprints > 24 than the 2019/20 (*p* < 0.001; ES: 0.49) and 2020/21 seasons (*p* < 0.05; ES: 0.16). When the Second Division was considered, in the 2021/22 season, teams also performed significantly more Nº. Sprints > 21 compared to the 2019/20 (*p* < 0.001; ES: 0.91), 2020/21 (*p* < 0.001; ES: 0.25), and 2022/23 seasons (*p* < 0.001; ES: 0.37) and more Nº. Sprints > 24 than the 2019/20 (*p* < 0.001; ES: 0.78) and 2020/21 seasons (*p* < 0.001; ES: 0.20). In general, trivial to moderate increases were seen in both soccer leagues for Nº. Sprints > 21 and Nº. Sprints > 24 variables between the 2019/20 and 2022/23 seasons (see Table S1).

**Figure 2:**
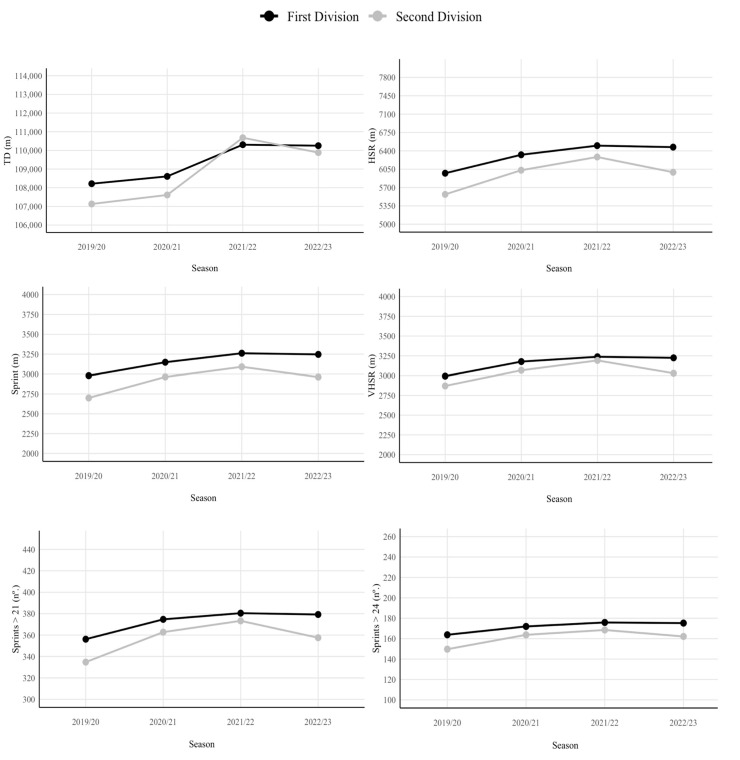
Evolution of match running performance over the seasons for each league. Note. m = meters; TD = total distance; HSR = high-speed running; VHSR = very high-speed running; Sprint = sprinting speed running distance.

Finally, Figure 2 shows the evolution of match running performance in the First and Second Divisions over the four seasons.

## 4. Discussion

The aim of the current study was twofold: (i) to compare match running performance between the top two competitive levels of Spanish professional soccer leagues and (ii) to analyze and compare the evolution of match running performance between the First and Second Spanish Divisions over four consecutive seasons (2019/20–2022/23). This study provides an updated understanding of match running performance in Spanish professional soccer leagues since previously conducted studies [13]. The main findings of the study revealed that (a) match running performance was significantly higher in the First Division than in the Second Division across all variables analyzed and (b) considering the evolution of both the First and Second Divisions, match running performance significantly increased in both leagues over the four seasons, with notably higher values during the 2021/22 and 2022/23 seasons.

Studies that have analyzed other European leagues (e.g., English, Spanish, and Norwegian leagues) have reported higher match physical demands in higher-standard leagues [12,13,38]. This study also found greater match running performance in the First Division compared to the Second Division. As hypothesized, our results showed higher TD, distances covered at high intensity, and the number of HSR efforts in the higher-standard leagues. Several factors could explain these findings. Firstly, one possible explanation might be the superior physical abilities of First Division players [39]. In this regard, previous research has indicated higher aerobic fitness and peak velocity in elite soccer players compared to sub-elite players [40,41]. Another possible explanation could be the playing styles employed in the First Division, which may demand higher match physical output [14,42,43]. However, the primary reason for these findings could be the effective playing time. Recent studies have reported that effective playing time is higher in the First Division than in the Second Division, which may influence differences in match running performance between standard leagues [44,45]. Specifically, when effective playing time is considered, the distance covered by teams appears similar across lower and higher competitive standards [42,46].

In addition, the analysis of match running performance by halves showed an increase in all variables during the second halves. Contrary to previous studies that reported a decrease in the second halves [13,47], our results showed an increase in both league standards during the second halves. These findings could be explained by the enhanced physical abilities of current soccer players due to more demanding training regimens in recent years, enabling players to sustain HSR efforts throughout matches [48]. Another possible explanation is the distance covered by substitutes during the second halves [49,50]. It is also noteworthy that since the 2019/20 season, five substitutions have been allowed, which may contribute to increased match running performance by teams during the second halves.

Concerning the evolution of match running performance between the First and Second Divisions over four consecutive seasons, we expected an increase in TD, distances covered at high intensity, and HSR efforts in both professional leagues. Overall, the results showed that all external load variables included in the analysis increased over the years. These findings align with recent studies reporting a progressive increase over seasons in match running performance in other European leagues, such as the English Premier League [19]. Similarly, in the Russian Premier League, TD, HSR and sprint distances, and maximal acceleration showed an average increase from the 2016–2017 season to the 2018–2019 season, although followed by a decrease in the subsequent season [18]. Consequently, the evolution of match running performance in the Spanish professional soccer leagues may be linked to higher physical demands in the coming years, contrasting with earlier research that reported a decrease in TD [3,4,13,17]. HSR distance also significantly increased over the years, consistent with previous studies conducted in Spanish professional soccer leagues [13], the Russian Premier League [18], and the English Premier League [19]. These findings reflect the ongoing evolution in modern soccer, where players are now trained to perform more high-intensity actions [48,51].

Several factors may explain these findings. Firstly, stoppages (e.g., Video Assistant Referee reviews) in soccer may allow for greater player recovery, enabling more sprints and increasing game intensity [16,46]. Another possible explanation is the current training focus, which has increased the emphasis on HSR to optimize player performance while minimizing injury risk [26]. Additionally, playing style may influence this evolution, with teams implementing advanced defensive lines, resulting in larger spaces and more high-intensity actions to exploit these areas. Finally, recent rule changes, such as the increase in the number of substitutions (from three to five), have likely impacted match running performance, increasing distances covered in professional soccer, so players who typically play entire matches are subjected to greater physical demands. In this context, non-starters may have contributed to the increased HSR distance covered by teams [49,50].

### 4.1. Limitations and Future Directions

Concerning the limitations of the current study, several aspects should be acknowledged as follows: (i) the analysis only considered total playing time, so effective playing time should be included in future studies to gain a more comprehensive understanding of actual match physical demands; (ii) future research should introduce the average minutes played by soccer players, differentiating between total playing time and effective time; (iii) various contextual variables that could influence match running performance, such as playing style [14], opponent quality [52], or team formation [53], were not considered; (iv) as soccer players are reaching increasingly higher speeds [23], speed thresholds above 28 km·h^−1^ should be included in future research; and (v) accelerations and decelerations should also be considered, as they are an integral part of the external load in soccer matches [54].

### 4.2. Practical Applications

Our findings have practical implications that may assist practitioners and strength and conditioning coaches in understanding the evolution of match running performance in Spanish professional soccer leagues. Firstly, the results indicate that modern soccer is becoming more demanding, so training approaches should adapt to current match physical demands to optimize player performance. Secondly, continuously and exhaustively monitoring match running performance should be essential for optimizing training periodization tailored to soccer players. In addition, practitioners should plan harder training sessions, including more HSR, VHSR, and sprint distance and high-intensity actions during mycrocicle by running-based drills or ball-based drills like transition games [55,56]. Tapering or short breaks during mycrocicle would also be necessary to assimilate higher intensity demands and avoid overreaching injuries [57]. These strategies could also serve as a methodology for injury prevention and potentially reduce injury rates among soccer players. Additionally, high-intensity training is essential to meet the high-intensity demands observed in the second halves of matches. Finally, sprint distance (i.e., >24 km·h^−1^) is gaining importance in soccer actions, warranting deeper analysis and the inclusion of sprint-specific training tasks in soccer practice [23].

## 5. Conclusions

The present study explains and compares match running performance differences between competitive standards over four consecutive seasons. Firstly, the data demonstrate that the distance covered and HSR efforts by soccer teams were significantly higher in the First Division compared to the Second Division. Secondly, the evolution of match running performance showed a significant increase in both leagues over the four seasons, with notably higher values during the 2021/22 season. To sum up, the evolution of match running performance in modern soccer should be considered from a training perspective to ensure players are prepared to meet current match physical demands. So, continuously monitoring changes in match physical demands in professional soccer should be essential.

## Figures and Tables

**Table 1:** Match running performance differences between leagues.

	First Division		Second Division		*p*
	Coeff	SE	Coeff	SE	
TD (m)	109,347	111	108,839	100	***
TD First Half (m)	54,498	59.56	54,206	53.91	***
TD Second Half (m)	54,848	69.90	54,632	63.27	*
HSR (m)	6318	20.5	5972	18.6	***
HSR First Half (m)	3120	11.9	2951	10.7	***
HSR Second Half (m)	3198	12.6	3021	11.4	***
VHSR (m)	3158	9.51	3041	8.61	***
VHSR First Half (m)	1563	5.45	1505	4.94	***
VHSR Second Half (m)	1596	5.73	1537	5.18	***
Sprint (m)	3159	12.6	2931	11.4	***
Sprint First Half (m)	1557	7.53	1447	6.82	***
Sprint Second Half (m)	1602	7.99	1484	7.23	***
Nº. Sprints > 21 (nº.)	373	1.01	357	0.91	***
Nº. Sprints > 21 First Half (nº.)	187	0.56	179	0.51	***
Nº. Sprints > 21 Second Half (nº.)	186	0.59	179	0.54	***
Nº. Sprints > 24 (nº.)	172	0.57	161	0.52	***
Nº. Sprints > 24 First Half (nº.)	85.6	0.34	80.3	0.30	***
Nº. Sprints > 24 Second Half (nº.)	86.1	0.35	80.8	0.32	***

Note. Coeff = coefficient; SE = standard error; m = meters; TD = total distance; HSR = high-speed running; VHSR = very high-speed running; Sprint = sprint speed running distance; * *p* < 0.05; *** *p* < 0.001.

**Table 2:** TD and HSR differences between seasons.

	Seasons	L1		*p*	L2		*p*
		Coeff	SE		Coeff	SE	
TD (m)	2019/20	108,215	212	c ***, d ***	107,129	194	c ***, d ***
	2020/21	108,608	211	c ***, d ***	107,612	190	c ***, d ***
	2021/22	110,305	211	a ***, b ***	110,678	190	a ***, b ***, d **
	2022/23	110,252	211	a ***, b ***	109,882	191	a ***, b ***, c **
TD First Half (m)	2019/20	54,194	116	c ***, d ***	53,627	107	c ***, d ***
	2020/21	54,068	116	c ***, d ***	53,568	104	c ***, d ***
	2021/22	54,906	116	a ***, b ***	55,068	105	a ***, b ***, d **
	2022/23	54,823	116	a ***, b ***	54,543	105	a ***, b ***, c **
TD Second Half (m)	2019/20	54,019	135	b **, c ***, d ***	53,498	123	b **, c ***, d ***
	2020/21	54,539	134	a **, c ***, d ***	54,044	121	a **, c ***, d ***
	2021/22	55,400	134	a ***, b ***	55,610	121	a ***, b ***
	2022/23	55,429	134	a ***, b ***	55,339	121	a ***, b ***
HSR (m)	2019/20	5973	39.4	b **, c ***, d ***	5569	36.1	b ***, c ***, d ***
	2020/21	6325	39.2	a **, c **, d **	6030	35.3	a ***, c ***
	2021/22	6499	39.3	a ***, b **	6282	35.4	a ***, b ***, d ***
	2022/23	6471	39.3	a ***, b **	5992	35.5	a ***, c ***
HSR First Half (m)	2019/20	2953	23.1	b ***, c ***, d ***	2753	21.1	b ***, c ***, d ***
	2020/21	3123	22.9	a ***, c **, d *	2970	20.7	a ***, c **
	2021/22	3209	23.0	a ***, b **	3108	20.7	a ***, b ***, d ***
	2022/23	3191	23.0	a ***, b **	2967	20.8	a ***, c ***
HSR Second Half (m)	2019/20	3019	24.6	b ***, c ***, d ***	2816	22.5	b ***, c ***, d ***
	2020/21	3202	24.5	a ***, c *, d *	3061	22.0	a ***, c **
	2021/22	3290	24.5	a ***, b *	3174	22.1	a ***, b **, d ***
	2022/23	3279	24.5	a ***, b *	3025	22.1	a ***, c ***

Note. Coeff = coefficient; SE = standard error; m = meters; TD = total distance; HSR = high-speed running; a = significant differences compared with 2019/20; b = significant differences compared with 2020/21; c = significant differences compared with 2021/22; d = significant differences compared with 2022/23; * *p* < 0.05; ** *p* < 0.01; *** *p* < 0.001.

**Table 3:** VHSR and sprint differences between seasons.

	Seasons	L1		*p*	L2		*p*
		Coeff	SE		Coeff	SE	
VHSR (m)	2019/20	2993	18.3	b ***, c ***, d ***	2870	16.7	b ***, c ***, d ***
	2020/21	3177	18.2	a ***, c *	3067	16.4	a ***, c ***
	2021/22	3238	18.2	a ***, b *	3192	16.4	a ***, b ***, d ***
	2022/23	3224	18.2	a ***	3030	16.5	a ***, c ***
VHSR First Half (m)	2019/20	1486	10.63	b ***, c ***, d ***	1421	9.73	b ***, c ***, d ***
	2020/21	1569	10.58	a ***, c *	1514	9.53	a ***, c ***
	2021/22	1600	10.59	a ***, b *	1577	9.54	a ***, b ***, d ***
	2022/23	1596	10.59	a ***	1504	9.57	a ***, c ***
VHSR Second Half (m)	2019/20	1507	11.13	b ***, c ***, d ***	1449	10.18	b ***, c ***, d ***
	2020/21	1608	11.07	a ***, c *	1553	9.98	a ***, c ***, d *
	2021/22	1638	11.09	a ***, b *	1615	9.99	a ***, b ***, d ***
	2022/23	1628	11.09	a ***	1526	10.02	a ***, b *, c ***
Sprint (m)	2019/20	2980	24.5	b ***, c ***, d ***	2699	22.4	b ***, c ***, d ***
	2020/21	3148	24.3	a ***, c **, d **	2963	21.9	a ***, c ***
	2021/22	3261	24.4	a ***, b **	3091	21.9	a ***, b ***, d ***
	2022/23	3246	24.4	a ***, b **	2962	22.0	a ***, c ***
Sprint First Half (m)	2019/20	1468	14.8	b ***, c ***, d ***	1333	13.5	b ***, c ***, d ***
	2020/21	1554	14.7	a ***, c **, d *	1455	13.2	a ***, c ***
	2021/22	1609	14.7	a ***, b **	1532	13.2	a ***, b ***, d ***
	2022/23	1595	14.7	a ***, b *	1463	13.3	a ***, c ***
Sprint Second Half (m)	2019/20	1512	15.7	b ***, c ***, d ***	1367	14.4	b ***, c ***, d ***
	2020/21	1594	15.6	a ***, c *, d *	1508	14.1	a ***, c *
	2021/22	1652	15.6	a ***, b *	1559	14.1	a ***, b *, d **
	2022/23	1651	15.6	a ***, b *	1499	14.1	a ***, c **

Note. Coeff = coefficient; SE = standard error; m = meters; VHSR = very high-speed running; Sprint = sprint speed running distance; a = significant differences compared with 2019/20; b = significant differences compared with 2020/21; c = significant differences compared with 2021/22; d = significant differences compared with 2022/23; * *p* < 0.05; ** *p* < 0.01; *** *p* < 0.001.

**Table 4:** Nº. Sprints > 21 and Nº. Sprints > 24 differences between seasons.

	Seasons	L1		*p*	L2		*p*
		Coeff	SE		Coeff	SE	
Nº. Sprints > 21 (nº.)	2019/20	356	1.92	b ***, c ***, d ***	335	1.76	b ***, c ***, d ***
	2020/21	375	1.91	a ***, c *	363	1.72	a ***, c ***, d *
	2021/22	380	1.91	a ***, b *	373	1.72	a ***, b ***, d ***
	2022/23	379	1.91	a ***	358	1.73	a ***, b *, c ***
Nº. Sprints > 21 First Half (nº.)	2019/20	179	1.09	b ***, c ***, d ***	168	1.00	b ***, c ***, d ***
	2020/21	187	1.08	a ***, c *, d *	181	0.98	a ***, c ***
	2021/22	190	1.08	a ***, b *	187	0.98	a ***, b ***, d ***
	2022/23	190	1.08	a ***, b *	179	0.98	a ***, c ***
Nº. Sprints > 21 Second Half (nº.)	2019/20	178	1.14	b ***, c ***, d ***	167	1.05	b ***, c ***, d ***
	2020/21	188	1.14	a ***	182	1.02	a ***, c ***, d *
	2021/22	190	1.14	a ***	187	1.02	a ***, b ***, d ***
	2022/23	189	1.14	a ***	179	1.03	a ***, b *, c ***
Nº. Sprints > 24 (nº.)	2019/20	164	1.11	b ***, c ***, d ***	150	1.01	b ***, c ***, d ***
	2020/21	172	1.10	a ***, c *, d *	164	0.99	a ***, c ***
	2021/22	176	1.10	a ***, b *	169	0.99	a ***, b ***
	2022/23	175	1.10	a ***, b *	162	1.00	a ***, c ***
Nº. Sprints > 24 First Half (nº.)	2019/20	81.7	0.66	b ***, c ***, d ***	74.7	0.60	b ***, c ***, d ***
	2020/21	85.8	0.65	a ***, c *	81.3	0.59	a ***, c **
	2021/22	87.6	0.65	a ***, b *	84.0	0.59	a ***, b **, d ***
	2022/23	87.4	0.65	a ***	81.0	0.59	a ***, c ***
Nº. Sprints > 24 Second Half (nº.)	2019/20	82.1	0.68	b ***, c ***, d ***	74.9	0.63	b ***, c ***, d ***
	2020/21	86.2	0.68	a ***, c *	82.4	0.61	a ***, c *
	2021/22	88.3	0.68	a ***, b *	84.5	0.61	a ***, b *, d ***
	2022/23	87.8	0.68	a ***	81.2	0.61	a ***, c ***

Note. Coeff = coefficient; SE = standard error; n = numbers; a = significant differences compared with 2019/20; b = significant differences compared with 2020/21; c = significant differences compared with 2021/22; d = significant differences compared with 2022/23; * *p* < 0.05; ** *p* < 0.01; *** *p* < 0.001.

## Data Availability

Restrictions apply to the availability of these data. Data were obtained from LaLiga and are available at https://www.laliga.es/en (accessed on 12 July 2023) with the permission of LaLiga.

## Supplementary Materials

The following supporting information can be downloaded at https://www.mdpi.com/article/10.3390/jfmk10010027/s1. Table S1: Effect size comparisons between four seasons for both leagues.
