# Supplementary material for: The Evolution of Match Running Performance in the Top Two Spanish Soccer Leagues: A Comparative Four-Season Study
Source: J Funct Morphol Kinesiol. 2025 Jan 10;10(1):27. doi: 10.3390/jfmk10010027 (PMC11755642; doi:10.3390/jfmk10010027)
Supplement: Supplementary file 1 [file jfmk-10-00027-s001.zip › jfmk-3368253-supplementary.pdf]

Table S1. Effect size comparisons between four seasons for both leagues.

Variables	Leagues	η2	d ($CI_{95\%}$)					
			2019/20 vs 2020/21	2019/20 vs 2021/22	2019/20 vs 2022/23	2020/21 vs 2021/22	2020/21 vs 2022/23	2021/22 vs 2022/23
TD (m)	First Division	.04	-.09 (-.19, .01)	-.47 (-.57, -.37)	-.46 (-.56, -.36)	-.38 (-.48, -.28)	-.37 (-.47, -.27)	.01 (-.09, .11)
TD 1st Half (m)		.02	.05 (-.05, .15)	-.29 (-.39, -.19)	-.26 (-.36, -.16)	-.34 (-.44, -.24)	-.31 (-.41, -.21)	.03 (-.07, .13)
TD 2nd Half (m)		.04	-.19 (-.29, -.08)	-.49 (-.59, -.39)	-.50 (-.61, -.40)	-.31 (-.41, -.21)	-.32 (-.42, -.22)	-.01 (-.11, .09)
HSR (m)		.06	-.41 (-.51, -.31)	-.61 (-.71, -.51)	-.58 (-.68, -.48)	-.20 (-.30, -.10)	-.17 (-.27, -.07)	.03 (-.07, .13)
HSR 1st Half (m)		.04	-.34 (-.44, -.24)	-.52 (-.62, -.41)	-.48 (-.58, -.38)	-.17 (-.27, -.07)	-.14 (-.24, -.04)	.04 (-.06, .14)
HSR 2nd Half (m)		.04	-.34 (-.45, -.24)	-.51 (-.61, -.41)	-.49 (-.59, -.39)	-.17 (-.27, -.07)	-.15 (-.25, -.05)	.02 (-.08, .12)
VHSR (m)		.05	-.44 (-.54, -.33)	-.58 (-.68, -.48)	-.55 (-.65, -.45)	-.15 (-.25, -.04)	-.11 (-.21, -.01)	.03 (-.07, .13)
VHSR 1st Half (m)		.03	-.34 (-.44, -.24)	-.46 (-.56, -.36)	-.45 (-.55, -.35)	-.13 (-.23, -.02)	-.11 (-.21, -.01)	.02 (-.09, .12)
VHSR 2nd Half (m)		.04	-.40 (-.50, -.30)	-.52 (-.62, -.41)	-.48 (-.58, -.38)	-.12 (-.22, -.02)	-.08 (-.18, .02)	.04 (-.06, .14)
Sprint (m)		.04	-.32 (-.42, -.22)	-.53 (-.63, -.43)	-.50 (-.61, -.40)	-.21 (-.31, -.11)	-.19 (-.29, -.08)	.03 (-.07, .13)
Sprint 1st Half (m)		.03	-.27 (-.37, -.17)	-.45 (-.55, -.35)	-.40 (-.50, -.30)	-.17 (-.28, -.07)	-.13 (-.23, -.03)	.04 (-.06, .15)
Sprint 2nd Half (m)		.03	-.24 (-.34, -.14)	-.41 (-.51, -.31)	-.41 (-.51, -.31)	-.17 (-.27, -.07)	-.17 (-.27, -.07)	.00 (-.10, .10)
Nº. Sprints > 21 (nº.)		.05	-.43 (-.53, -.33)	-.57 (-.67, -.46)	-.54 (-.64, -.43)	-.13 (-.24, -.03)	-.11 (-.21, .00)	.03 (-.07, .13)
Nº. Sprints > 21 1st Half (nº.)		.04	-.34 (-.44, -.24)	-.48 (-.58, -.38)	-.46 (-.56, -.36)	-.14 (-.25, -.04)	-.13 (-.23, -.03)	.02 (-.08, .12)
Nº. Sprints > 21 2nd Half (nº.)		.04	-.41 (-.51, -.30)	-.49 (-.60, -.39)	-.46 (-.56, -.36)	-.09 (-.19, .01)	-.06 (-.16, .04)	.03 (-.07, .13)
Nº. Sprints > 24 (nº.)		.04	-.33 (-.44, -.23)	-.49 (-.60, -.39)	-.47 (-.57, -.37)	-.16 (-.26, -.06)	-.13 (-.23, -.03)	.03 (-.07, .13)
Nº. Sprints > 24 1st Half (nº.)		.03	-.28 (-.38, -.18)	-.41 (-.51, -.31)	-.39 (-.50, -.29)	-.13 (-.23, -.03)	-.11 (-.21, -.01)	.02 (-.09, .12)
Nº. Sprints > 24 2nd Half (nº.)		.03	-.27 (-.37, -.17)	-.41 (-.51, -.30)	-.38 (-.48, -.28)	-.14 (-.24, -.04)	-.11 (-.21, -.01)	.03 (-.07, .13)
TD (m)	Second Division	.10	-.11 (-.20, -.02)	-.79 (-.88, -.69)	-.61 (-.70, -.52)	-.68 (-.77, -.59)	-.50 (-.59, -.41)	.18 (.09, .27)
TD 1st Half (m)		.06	.02 (-.07, .12)	-.59 (-.68, -.50)	-.38 (-.47, -.28)	-.62 (-.71, -.52)	-.40 (-.49, -.31)	.22 (.12, .31)
TD 2nd Half (m)		.09	-.19 (-.28, -.10)	-.74 (-.83, -.65)	-.64 (-.74, -.55)	-.55 (-.64, -.46)	-.45 (-.54, -.36)	.09 (.00, .19)
HSR (m)		.08	-.55 (-.64, -.46)	-.85 (-.95, -.76)	-.51 (-.60, -.41)	-.30 (-.39, -.21)	.05 (-.04, .14)	.35 (.26, .44)
HSR 1st Half (m)		.06	-.44 (-.53, -.35)	-.72 (-.81, -.63)	-.43 (-.53, -.34)	-.28 (-.37, -.19)	.01 (-.08, .10)	.29 (.20, .38)
HSR 2nd Half (m)		.06	-.48 (-.57, -.39)	-.70 (-.80, -.61)	-.41 (-.50, -.32)	-.22 (-.31, -.13)	.07 (-.02, .16)	.29 (.20, .38)
VHSR (m)		.07	-.49 (-.58, -.40)	-.80 (-.89, -.71)	-.40 (-.49, -.30)	-.31 (-.40, -.22)	.09 (.00, .18)	.40 (.31, .49)
VHSR 1st Half (m)		.05	-.39 (-.48, -.30)	-.65 (-.75, -.56)	-.35 (-.44, -.26)	-.26 (-.35, -.17)	.04 (-.05, .13)	.30 (.21, .40)
VHSR 2nd Half (m)		.05	-.42 (-.51, -.33)	-.67 (-.77, -.58)	-.31 (-.40, -.22)	-.25 (-.34, -.16)	.11 (.02, .20)	.36 (.27, .45)

Sprint (m)	.07	-.51 (-.60, -.42)	-.76 (-.85, -.67)	-.51 (-.60, -.42)	-.25 (-.34, -.16)	.00 (-.09, .09)	.25 (.16, .34)
Sprint 1st Half (m)	.05	-.39 (-.49, -.30)	-.64 (-.73, -.54)	-.42 (-.51, -.32)	-.24 (-.33, -.15)	-.02 (-.12, .07)	.22 (.13, .31)
Sprint 2nd Half (m)	.05	-.44 (-.53, -.34)	-.60 (-.69, -.50)	-.41 (-.50, -.32)	-.16 (-.25, -.07)	.03 (-.06, .12)	.19 (.10, .28)
Nº. Sprints > 21 (nº.)	.10	-.66 (-.75, -.57)	-.91 (-1.01, -.82)	-.54 (-.63, -.45)	-.25 (-.34, -.16)	.12 (.03, .21)	.37 (.28, .46)
Nº. Sprints > 21 1st Half (nº.)	.07	-.54 (-.63, -.45)	-.77 (-.86, -.67)	-.46 (-.55, -.36)	-.23 (-.32, -.14)	.08 (-.01, .17)	.31 (.22, .40)
Nº. Sprints > 21 2nd Half (nº.)	.08	-.60 (-.69, -.51)	-.80 (-.90, -.71)	-.47 (-.56, -.38)	-.20 (-.29, -.11)	.13 (.04, .22)	.33 (.24, .42)
Nº. Sprints > 24 (nº.)	.08	-.58 (-.67, -.49)	-.78 (-.87, -.68)	-.51 (-.61, -.42)	-.20 (-.29, -.11)	.07 (-.02, .16)	.26 (.17, .35)
Nº. Sprints > 24 1st Half (nº.)	.05	-.46 (-.55, -.36)	-.64 (-.73, -.55)	-.43 (-.52, -.34)	-.19 (-.28, -.09)	.02 (-.07, .11)	.21 (.12, .30)
Nº. Sprints > 24 2nd Half (nº.)	.05	-.51 (-.60, -.42)	-.65 (-.74, -.56)	-.42 (-.52, -.33)	-.14 (-.23, -.05)	.09 (-.01, .18)	.23 (.14, .32)

Note. m = meters; TD = Total Distance; HSR = High Speed Running; VHSR = Very High-Speed Running; Sprint = Sprint Speed Running Distance.
